# Burden of oral diseases predicts development of excess weight in early adolescence: a 2-year longitudinal study

**DOI:** 10.1007/s00431-024-05663-8

**Published:** 2024-07-03

**Authors:** Sohvi Lommi, Jukka Leinonen, Pirkko Pussinen, Jussi Furuholm, Kaija-Leena Kolho, Heli Viljakainen

**Affiliations:** 1 Folkhälsan Research Center, Helsinki, Finland; 2 Faculty of Medicine, University of Helsinki, Helsinki, Finland; 3 Institute of Dentistry, University of Eastern Finland, Kuopio, Finland; 4 Oral and Maxillofacial Diseases, University of Helsinki, Helsinki, Finland; 5 Children’s Hospital, University of Helsinki and Helsinki University Hospital (HUS), Helsinki, Finland; 6 Faculty of Medicine and Health Technology, Tampere University, Tampere, Finland

**Keywords:** Caries, Cohort studies, Gingivitis, Obesity, Pediatrics

## Abstract

Dental caries, gingivitis, and excess weight are highly prevalent, interconnected chronic conditions. The association of oral health with the development of adiposity among children is sparsely addressed. We examined the association of oral health to the development of excess weight and central obesity in early adolescence during a 2-year follow-up period. This prospective study was conducted with 2702 children aged 9–12 years at baseline from the Finnish Health in Teens study. Their weight development was followed up for 2 years. Body mass index with age- and sex-specific cut-offs and the waist–height ratio indicated weight status and central obesity. Oral health data (caries experience and gingivitis/calculus) were collected from outpatient records of public dental services. Having both caries experience and gingivitis/calculus was considered burden of oral diseases. Of the sample, 74% were caries-free but 70% exhibited gingivitis and/or calculus, and 20% had both caries experience and gingivitis/calculus. During the follow-up period, 5.3% (*n* = 124) and 4.7% (*n* = 118) of the children became overweight/obese or centrally obese, respectively. Having both caries experience and gingivitis/calculus associated with the development of excess weight in a fully adjusted model (HR 1.75, 95% CI 1.03–2.97) but not of central obesity. Caries experience or gingivitis/calculus alone did not associate with adiposity development.

*Conclusion*: Having burden of oral diseases without excess weight at early adolescence could imply future weight gain; thus, normal-weight individuals with both caries experience and gingivitis/calculus could be targeted with preventive measures. Our findings warrant further research to explore whether oral diseases and the development of obesity merely share risk factors or if their relationship is of causal nature.

**Table:** 

**What is Known:** • *Association of excess weight with caries experience and gingivitis is known to exist both cross-sectionally and longitudinally in children and adolescents.*
**What is New:** • *Burden of oral diseases, that is, having both caries experience and gingivitis/calculus, was associated with becoming overweight or obese 2 years later during early adolescence.* • *Normal-weight individuals with burden of oral diseases at early adolescence could be targeted with preventive measures against excess weight gain.*

**Supplementary Information:**

The online version contains supplementary material available at 10.1007/s00431-024-05663-8.

## Introduction

Excess weight, dental caries, and gingivitis are common conditions among children and adolescents with an early onset. Among European children and adolescents, nearly one-third are overweight or obese [1] and 20–60% are affected by caries already by the age of 6 [2]. Although fewer studies exist on prevalence of gingivitis in children and adolescents, it seems to be as prevalent as caries [3]. For instance, 60% of 12-year-olds suffered from gingivitis, which is also a tell-tale sign of poor oral hygiene [4]. The association of excess weight with caries in childhood has been studied quite extensively both in cross-sectional and longitudinal studies, as a recent overview of systematic reviews shows [5]. However, results are rather inconclusive and conflicting, which could partially be due to different populations, assessment methods, and confounders. Moreover, a positive association of overweight/obesity with periodontal conditions such as calculus, periodontitis, and gingivitis has been observed [6, 7]. Only some studies have examined the relationship of oral health to subsequent weight development. For example, high caries experience in 15-year-old Chinese children predicted central obesity but not excess weight 3 years later [8]. These findings were not replicated among younger children [8, 9]. Among Finns, caries experience at childhood associated with waist circumference in adulthood [10]. Indeed, signs of poor oral health during childhood may predict obesity or metabolic disturbances during adulthood [10, 11]. Yet, longitudinal evidence is still lacking on the relationships of childhood caries experience and gingivitis to excess weight and central obesity, the last two being typical precursors for cardiometabolic disturbances in adulthood [12, 13].

Caries and gingivitis are both biofilm-mediated diseases in which aberrant oral microbiota plays a key role [14, 15]. The link to obesity has been hypothesized through the oral microbiota, with possible mechanisms such as low-grade systemic inflammation and increased oxidative stress induced by periodontal disease [16]. Recent research has unraveled the role of gut microbiome in local and systemic diseases, including obesity [17]. Oral cavity works as a microbial reservoir for the gut microbiome, and through ingested saliva, transmission of oral bacteria to the gut is extensive even in healthy individuals [18]. Gut microbes have been proposed to affect eating behaviors through manipulating satiety [19], while oral bacteria and their metabolism could impact taste perceptions and preferences in the oral cavity [20, 21], possibly leading to altered food consumption. These mechanisms provide a plausible explanation for the link between oral health and development of obesity. Then again, oral diseases could be comorbidities or complications of obesity; an opposite direction of the association has been suggested such as obesity modifying the oral microbiota and thus leading to oral diseases [22]. Excess weight and poor oral health share several risk factors including poor diet, excessive sugar consumption considered as the most prominent one [23–25]. The interconnected, multifactorial nature of these conditions and their shared risk factors complicate the understanding of the directions of the associations.

In this research report, we aimed to examine the longitudinal relationships of caries experience, gingivitis/calculus, and the burden of these two diseases with the risk of developing excess weight or central obesity during a 2-year follow-up period among Finnish children aged on average 11 years at baseline.

## Methods

This longitudinal study utilized data from the Finnish Health in Teens (Fin-HIT) cohort, which includes over 11,000 Finnish children mostly aged 9–12 at enrolment. The Fin-HIT baseline data collection at study visits was conducted in 2011–2014 primarily through school-based recruitment. The first active follow-up was completed in 2015 and 2016, when children participated at home. The participation rate at follow-up was 54%. In addition, we collected oral health data from the Register of Primary Health Care visits (Avohilmo) maintained by the Finnish Institute for Health and Welfare [26]. The Fin-HIT study adhered to the Declaration of Helsinki, and the study protocol was approved by the Coordinating Ethics Committee of the Hospital District of Helsinki and Uusimaa (169/13/03/00/10). Children and one guardian of each child provided their written informed consent. We followed the STROBE guidelines when reporting the findings [27]. In the present study, children with data on sex, age, maternal socioeconomic status, diet, physical activity, oral health, and baseline and follow-up measures of height, weight, and waist circumference were included (*n* = 2702). The study flowchart is available in the Supplementary material (Fig. S1).

### Measurements

#### Caries experience and periodontal health

Caries experience and periodontal health were clinically examined by a dentist or dental hygienist at a regular dental examination provided in a community dental clinic. We included only children whose dental examination was conducted a maximum of 365 days prior or after the Fin-HIT baseline data collection (median difference 8 days, IQR -120–108), with complete records on oral health variables. Based on the numbers of decayed, missing, and filled teeth (DMFT) in the permanent dentition, we calculated the DMFT index to indicate caries experience. Participants who had more than eight missing teeth were excluded due to the likely misregistration of primary teeth as missing. However, we additionally calculated the DFT index without the variable for missing teeth. The DFT index was used only in the supplementary analyses. Based on the DMFT index, we categorized children into two groups: absence of caries experience (DMFT = 0) and presence of caries experience (DMFT > 0). The DFT index was categorized in a similar manner. In addition, we estimated periodontal health using the community periodontal index for treatment needs (CPITN) [28] and categorized children into two groups: healthy (CPITN = 0) and with gingivitis/calculus (CPITN > 0). In addition, to examine the burden of oral diseases, we categorized the participants into those without caries experience and gingivitis/calculus (healthy), those with either caries experience or gingivitis/calculus, and those with both caries experience and gingivitis/calculus.

#### Anthropometrics

At baseline, fieldworkers measured children’s height, weight, and waist circumference during a school day in a standardized manner. At follow-up, we sent measuring tapes to the participants with instructions to the caregivers to measure their child’s height, waist circumference, and weight. The home-based measurements were deemed sufficiently accurate for epidemiological studies [29]. We calculated the body mass index (BMI) according to age- and sex-specific guidelines from the International Obesity Task Force [30] and categorized children as thin, normal weight, overweight, or obese. In the regression analysis (described below), we grouped overweight and obese together (= excess weight). Furthermore, to indicate central obesity, we calculated the waist–height ratio (WHtR) by dividing waist circumference by height and then categorized children as without (WHtR < 0.5) or with central obesity (WHtR ≥ 0.5).

#### Covariates

We estimated the weekly consumption frequency of sugary foods and drinks (sweet treats) as well as that of fruits, berries, and vegetables (plants) at baseline through summary scores (sweet treat index (STI) and plant consumption index (PCI)) based on a self-administered food frequency questionnaire, as described in detail elsewhere [31, 32]. We assessed the weekly leisure-time physical activity in hours at baseline based on a self-reported questionnaire. Age and sex were retrieved from the National Population Information System at the Population Register Center. Maternal occupation at the time of the child’s birth was used to determine socioeconomic status (SES), obtained from the Medical Birth Register from the National Institute for Health and Welfare [33]. Mothers were categorized as upper-level employees, lower-level employees, manual workers, students, or other (unemployed, stay-at-home mothers, and others).

### Statistical methods

We calculated descriptive statistics as means and standard deviations (SD), or as counts and percentages (%), and tested differences in means or proportions between categories using independent samples *t*-test or chi-square test, respectively. To examine the relationships between caries experience, gingivitis/calculus, and development of excess weight (= overweight or obesity), central obesity or both, we employed the multi-variable Cox regression analysis, considering the time between the baseline and the follow-up anthropometric measurements to produce hazard ratios (HRs) with 95% confidence intervals (CIs). We conducted both a crude regression analysis (model 1) and one adjusted for age, sex, maternal SES, sweet treat and plant consumption, and physical activity as well as periodontal health status or caries experience (model 2). Children who had excess weight or central obesity at study baseline were excluded from the analyses on developing the respective diseases. In a similar manner, we conducted supplementary analyses using the DFT index as the indicator of caries experience. We used SPSS statistics software version 29 (IBM Corp., Chicago, IL, USA) and set the level of statistical significance to *p* < 0.05.

## Results

### Characteristics of the sample

Children were on average 11.2 (SD 0.8) years old at baseline, and the mean follow-up time for weight development was 2.3 (0.3) years (Table 1). Of the children, 74% had no caries experience and 31% had neither gingivitis nor calculus recorded at the time of the dental examination (Tables 1 and 2). The mean DMFT was 0.6 (1.3). Table 1 shows the characteristics of the sample by caries experience and Table 2 by periodontal health status. Children with caries experience were more frequently overweight or obese at baseline and at follow-up and centrally obese at follow-up than those without caries experience (Table 1). Children with gingivitis/calculus were more frequently centrally obese at follow-up than those without gingivitis/calculus (Table 2). Of the sample, 25% had neither caries experience nor gingivitis/calculus, 56% had either caries experience or gingivitis/calculus, and 20% had burden of oral diseases, that is, both caries experience and gingivitis/calculus.

**﻿Table 1:** Participant characteristics by caries experience as means with standard deviations (SD) unless otherwise stated

			Caries experience				
	Total (*n* = 2702)		No (*n* = 1992)		Yes (*n* = 710)		*p*^a^
Sex, *n* (%)							0.849
Girl	1443	(53.4)	1066	(53.5)	377	(53.1)	
Boy	1259	(46.6)	926	(46.5)	333	(46.9)	
Maternal SES, *n* (%)							< 0.001
Upper-level employee	935	(34.6)	720	(36.1)	215	(30.3)	
Lower-level employee	1069	(39.6)	791	(39.7)	278	(39.2)	
Manual worker	257	(9.5)	171	(8.6)	86	(12.1)	
Student	255	(9.4)	190	(9.5)	65	(9.2)	
Other	186	(6.9)	120	(6.0)	66	(9.3)	
Periodontal health status, *n* (%)							< 0.001
Good	840	(31.1)	661	(33.2)	179	(25.2)	
Gingivitis/calculus	1862	(68.9)	1331	(66.8)	531	(74.8)	
*Baseline*							
Age (y)	11.2	(0.8)	11.1	(0.8)	11.3	(0.7)	< 0.001
STI, times/week	8.3	(7.2)	8.2	(7.3)	8.7	(7.0)	0.148
PCI, times/week	14.1	(8.7)	14.2	(8.7)	13.8	(8.8)	0.271
PA, hours/week	6.6	(2.8)	6.6	(2.8)	6.8	(2.8)	0.120
Weight status, *n* (%)							0.037
Thinness	354	(13.1)	276	(13.9)	78	(11.0)	
Normal weight	1997	(73.9)	1476	(74.1)	521	(73.4)	
Overweight	294	(10.9)	201	(10.1)	93	(13.1)	
Obesity	57	(2.1)	39	(2.0)	18	(2.5)	
Central obesity, *n* (%)							
No	2510	(92.9)	1868	(93.8)	642	(90.4)	0.003
Yes	192	(7.1)	124	(6.2)	68	(9.6)	
*Follow-up*							
Age (y)	13.5	(1.0)	13.5	(1.0)	13.6	(0.9)	< 0.001
Weight status, *n* (%)							0.036
Thinness	221	(8.2)	164	(8.2)	57	(8.0)	
Normal weight	2109	(78.1)	1574	(79.0)	535	(75.4)	
Overweight	325	(12.0)	218	(10.9)	107	(15.1)	
Obesity	47	(1.7)	36	(1.8)	11	(1.5)	
Central obesity, *n* (%)							0.073
No	2461	(91.1)	1826	(91.7)	635	(89.4)	
Yes	241	(8.9)	166	(8.3)	75	(10.6)	

^a^Result from Pearson’s chi-square test or independent samples *t*-test

PA, physical activity; PCI, plant consumption index (summary score of fruits, berries, and vegetables); SES, socioeconomic status; STI, sweet treat index (summary score of sweet treats)

**Table 2:** Participant characteristics by periodontal health status as means with standard deviations (SD) unless otherwise stated

			Periodontal health status				
	Total (*n* = 2702)		Healthy (*n* = 840)		Gingivitis/calculus (*n* = 1862)		*p*^a^
Sex, *n* (%)							< 0.001
Girl	1443	(53.4)	498	(59.3)	945	(50.8)	
Boy	1259	(46.6)	342	(40.7)	917	(49.2)	
Maternal socioeconomic status, *n* (%)							0.537
Upper-level employee	935	(34.6)	305	(36.3)	630	(33.8)	
Lower-level employee	1069	(39.6)	326	(38.8)	743	(39.9)	
Manual worker	257	(9.5)	84	(10.0)	173	(9.3)	
Student	255	(9.4)	71	(8.5)	184	(9.9)	
Other	186	(6.9)	54	(6.4)	132	(7.1)	
Caries experience, *n* (%)							< 0.001
No	1992	(73.7)	661	(78.7)	1331	(71.5)	
Yes	710	(26.3)	179	(21.3)	531	(28.5)	
*Baseline*							
Age (y)	11.2	(0.8)	11.2	(0.7)	11.2	(0.8)	0.704
STI, times/week	8.3	(7.2)	7.9	(6.3)	8.6	(7.5)	0.014
PCI, times/week	14.1	(8.7)	14.2	(8.5)	14	(8.8)	0.610
PA, hours/week	6.6	(2.8)	6.6	(2.8)	6.6	(2.8)	0.798
Weight status, *n* (%)							0.458
Thinness	354	(13.1)	110	(13.1)	244	(13.1)	
Normal weight	1997	(73.9)	633	(75.4)	1364	(73.3)	
Overweight	294	(10.9)	83	(9.9)	211	(11.3)	
Obesity	57	(2.1)	14	(1.7)	43	(2.3)	
Central obesity, *n* (%)							0.551
No	2510	(92.9)	784	(93.3)	1726	(92.7)	
Yes	192	(7.1)	56	(6.7)	136	(7.3)	
*Follow-up*							
Age (y)	13.5	(1.0)	13.6	(0.9)	13.5	(1.0)	0.059
Weight status, *n* (%)							0.342
Thinness	221	(8.2)	75	(8.9)	146	(7.8)	
Normal weight	2109	(78.1)	659	(78.5)	1450	(77.9)	
Overweight	325	(12.0)	96	(11.4)	229	(12.3)	
Obesity	47	(1.7)	10	(1.2)	37	(2.0)	
Central obesity, *n* (%)							0.030
No	2461	(91.1)	780	(92.9)	1681	(90.3)	
Yes	241	(8.9)	60	(7.1)	181	(9.7)	

^a^Result from Pearson’s chi-square test or independent samples *t*-test

PA, physical activity; PCI, plant consumption index (summary score of fruits, berries, and vegetables); SES, socioeconomic status; STI, sweet treat index (summary score of sweet treats)

### Oral health and the development of excess weight and central obesity

**Fig. 1:**
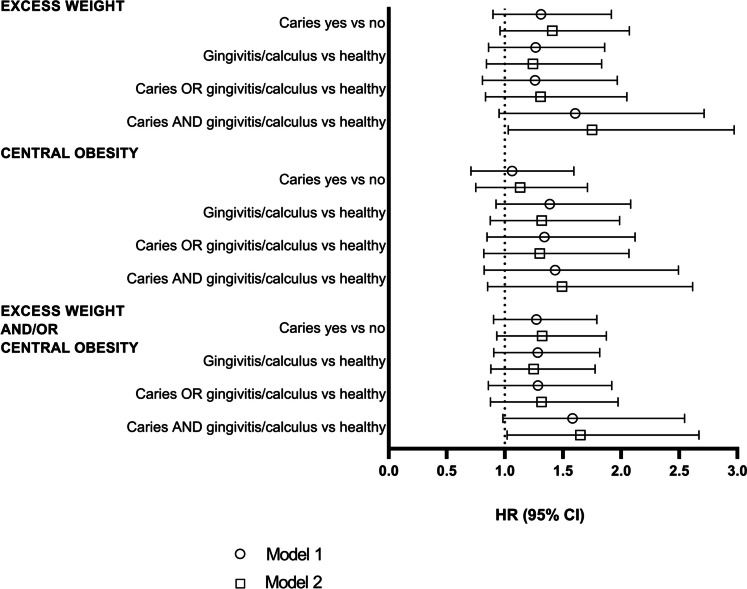
Associations of having caries experience, gingivitis/calculus, and/or both with development of excess weight and/or central obesity shown as hazard ratios (HR) with 95% confidence intervals (CI). Model 1 = crude, model 2 = variables included in the model were age at baseline, sex, maternal socioeconomic status, sweet treat and plant consumption at baseline, and caries experience or periodontal health status, respectively

Among the 2351 children who were either thin or normal weight at baseline, 5.3% (*n* = 124) developed excess weight by the end of the follow-up period. Similarly, among the 2510 children who had no central obesity at baseline, 4.7% (n = 118) developed central obesity. Among the 2334 children who had neither excess weight nor central obesity at baseline, 6.6% (*n* = 153) developed either excess weight or central obesity or both. Figure 1 shows the results of the Cox regression analysis for the longitudinal associations of having caries experience, gingivitis/calculus, and burden of oral diseases with developing excess weight and/or central obesity. We observed an association between the burden of oral diseases and an increased risk of development of excess weight (adjusted HR 1.75, 95% CI 1.03–2.97) and of development of excess weight and/or central obesity (1.65, 1.02–2.67), but not with the development of central obesity (1.49, 0.85–2.62). No association was detected for caries experience alone with the development of excess weight (1.31, 0.90–1.92), central obesity (1.13, 0.75–1.71), or excess weight and/or central obesity (1.32, 0.93–1.87). Similarly, we found no association between gingivitis/calculus and adiposity measures (1.24, 0.84–1.83; 1.32, 0.87–1.99; and 1.25, 0.88–1.78, respectively) or between having either caries experience or gingivitis/calculus and adiposity measures (1.31, 0.83–2.05; 1.30, 0.82–2.07; and 1.32, 0.88–1.97). The results using the DFT index as the indicator of caries experience followed similar patterns, although the associations between burden of oral diseases and anthropometrics were no longer statistically significant (Table S1).

## Discussion

In this large cohort of school-aged Finnish children, the burden of oral diseases was associated with increased risk of developing excess weight. Importantly, the association was independent of socioeconomic status, diet, and physical activity, suggesting a direct or indirect role of oral diseases in weight gain. Although non-significant, caries experience and gingivitis/calculus separately displayed slightly lower effect sizes with the same direction, thus increasing credibility of the findings. To our knowledge, this is a novel finding in this age group.

In previous studies, both caries and periodontal diseases have been associated with excess weight and central obesity in children [5, 6, 34, 35]. Here, we observed an increased risk between the burden of oral diseases (that is, having both caries experience and gingivitis/calculus) and developing excess weight during early adolescence, which may be a clinically relevant finding. In an older Finnish cohort, poor oral health in childhood (measured in 1980) associated with increases in BMI and waist circumference alongside other cardiometabolic conditions and risk factors such as a metabolic syndrome, increased blood pressure, and adverse lipid profiles during follow-up to adulthood [10, 11]. Diet and physical activity were not considered as confounders in those studies. However, caries alone was not associated with the development of BMI in that study, but initial weight status was not considered in the modeling. In a study on Chinese adolescents, a high level of caries experience predicted the development of central obesity in a 3-year follow-up period during late adolescence, but not during early adolescence [8]. In a 7-year follow-up study conducted in England, caries in early school-age was not associated with excess weight at adolescence [9]. That study did not consider the baseline weight status nor any health behaviors as covariates. In our study, the significant association between the burden of oral diseases and adiposity emerged after adjusting for covariates, e.g., shared risk factors. Then again, the estimates of the association were only marginally affected by the adjustment. The mean follow-up time of 2.3 years in our study was rather short, although it partially covers the quick growth stage from late childhood through early adolescence. With a longer follow-up time, the number of participants who developed excess adiposity may have been larger, likely strengthening the relationship we observed here.

Finns comprise a low caries population, which was evident in our study. Overall, 74% of children had no caries experience in permanent dentition, which is higher than the 61% found in a national sample of 12-year-olds in 2019 [36]. Then again, only one-third of the participants were free of gingivitis or dental calculus. Every fifth participant had both caries experience and gingivitis/calculus. The rather good oral health of our participants is likely explained by good oral hygiene and free-of-charge dental care for minors [37]. Fluoride toothpaste is commonly used in Finland [38], and 68% of Finnish fourth and fifth graders brushed their teeth twice daily [39]. We dichotomized the DMFT variable as the group sizes would otherwise be very uneven and small, hampering statistical power. This limited us from focusing on those children with multiple cavities, warranting further research in pediatric populations with a high number of cavities. Similarly, we dichotomized the CPITN variable. Deepened periodontal pockets (CPITN > 2) are rare in children [40] which limits the analysis to gingivitis and calculus. In the dental examination, the highest value in each sextant was reported, and the highest value of all the sextants determined the final CPITN value, meaning that those with the value of 2 were assigned as having calculus although they simultaneously could have had the value of 1 (gingivitis). We acknowledge that calculus is not a sign of gingivitis per se, yet they correlate [41]; thus, calculus was used as a proxy for gingivitis. Although calculus is not considered a disease, we used the word “disease” collectively for caries experience and gingivitis/calculus.

The strength of this study lies in the substantial number of children for whom data on a multitude of oral health and anthropometric measurements were available. Oral health data was obtained from a national register. However, DMFT and CPITN scores are relatively crude markers of caries experience and gingivitis/calculus and may be to some extent vulnerable to subjectivity of the professional. We limited our sampling to the dental check-ups performed by dentist or dental hygienist to overcome a part of the subjectivity. Nevertheless, the dental examinations between examiners were not calibrated. Moreover, as we cannot be certain that the teeth registered as missing were in fact unerupted permanent teeth, we repeated the analyses using the DFT index. These results were in line with the DMFT results although they did not reach statistical significance. The dental examinations were performed within 1 year before or after the Fin-HIT baseline data collection. Although some variation in the timing of the dental examinations existed, they took place before the follow-up on anthropometric measures, supporting temporal association.

We adjusted the analysis for self-reported baseline consumption of sweet treats and plants as well as for leisure-time physical activity as potential confounders possibly explaining the association to adiposity. In particular, the role of sugar is relevant and should be considered when examining the relationship between caries and excess weight [42]. The primary dietary determinant of caries is frequent consumption of sugar that cariogenic bacteria ferment in the oral cavity, leading to acidification of the dental biofilm and eventually to caries lesions if not controlled by remineralization enhancing activities especially tooth brushing with fluoride toothpaste [14]. In contrast, for youth obesity outcomes, the evidence of sugar seems to limit to sugar-sweetened beverages [43]. In our study, sweet treat consumption did not associate with anthropometric outcomes (data not shown), but regardless, residual confounding may exist. In addition, the Fin-HIT cohort likely reflects a sample with a slightly higher SES compared with the overall Finnish population [44] which may indicate a more health-conscious sample with lower rates of excess weight and better oral health compared with socially deprived samples. Loss to follow-up is common in prospective studies and was witnessed here as 46% of the initial cohort missed the follow-up data collection. Of the participants with oral health data available, 59% were either lost to follow-up or excluded due to missing questionnaire or anthropometric data. This group exhibited higher proportion of excess weight, central obesity, caries experience, and burden of oral diseases than those who were included in the current sample (Table S2). They also had lower maternal SES. Taken together, these limitations may decrease the generalizability of our findings to the broader population. However, given the direction of our findings, the associations we observed would probably be stronger in a more representative sample. In addition to the large proportion of participants lost to follow-up, a further limitation might be the rather low number of participants who developed excess weight or central obesity. This can result in an underpowered study and type II error. However, type II error means not rejecting the null hypothesis when it in fact is false. Even though not statistically significant, all hazard ratios in our analysis pointed toward a positive association of oral diseases with developing excess weight. This suggests that findings should be replicated with larger sample sizes or longer follow-up times. Moreover, we were limited to the use of maternal occupation at the time of child’s birth as the indicator for SES. Parental education and income levels might be more informative factors to express family’s SES than occupation alone.

To conclude, only a quarter of participants had caries experience whereas gingivitis/calculus were prevalent in two-thirds of the sample. Every fifth participant had both caries experience and gingivitis/calculus. The burden of having both caries and gingivitis/calculus predicted the development of excess weight but not that of central obesity when moving from late childhood to early adolescence. Having burden of oral diseases without excess weight in early adolescence could imply future weight gain and, thus, could be used to identify individuals at risk of gaining excess weight. Oral diseases and excess weight share risk factors, highlighting that our findings warrant further research to explore whether oral diseases and weight gain merely share risk factors or if their relationship is of causal nature.

### Supplementary Information

Below is the link to the electronic supplementary material.

Supplementary file1 (DOCX 104 KB)

## Data Availability

The datasets used and analyzed during the current study are available from the corresponding author on reasonable request.

## Abbreviations

BMI: Body mass index
CI: Confidence interval
DFT: Decayed and missing teeth
DMFT: Decayed, missing, and filled teeth
HR: Hazard ratio
SES: Socioeconomic status
WHtR: Waist–height ratio
