# Supplementary material for: Burden of oral diseases predicts development of excess weight in early adolescence: a 2-year longitudinal study
Source: Eur J Pediatr. 2024 Jul 3;183(9):4093–101. doi: 10.1007/s00431-024-05663-8 (PMC11322208; doi:10.1007/s00431-024-05663-8)
Supplement: Supplementary file 1 — Supplementary file1 (DOCX 104 KB) [file 431_2024_5663_MOESM1_ESM.docx]

**Supplementary material**

**Burden of oral diseases predicts development of excess weight in early adolescence: a two-year longitudinal study**

Sohvi Lommi^1,2^, Jukka Leinonen^3^, Pirkko Pussinen^3,4^, Jussi Furuholm^4^, Kaija-Leena Kolho^5,6^, Heli Viljakainen^1,2^

^1^Folkhälsan Research Center, Helsinki, Finland

^2^Faculty of Medicine, University of Helsinki, Helsinki, Finland

^3^Institute of Dentistry, University of Eastern Finland, Kuopio, Finland

^4^Oral and Maxillofacial Diseases, University of Helsinki, Finland

^5^Children’s Hospital, University of Helsinki and Helsinki University Hospital (HUS), Helsinki, Finland

^6^Faculty of Medicine and Health Technology, Tampere University, Tampere, Finland

**Corresponding author:**

Heli Viljakainen

heli.viljakainen@helsinki.fi

**
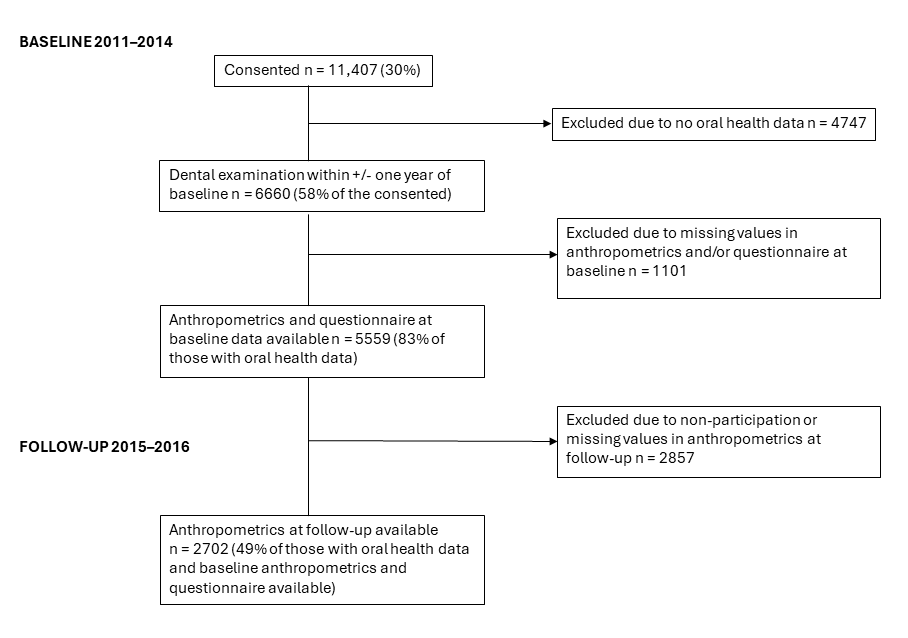
**

**Figure S1** Flowchart of the study

**Table S1** Associations of having caries experience (measured with the DFT index), gingivitis/calculus, and/or both with development of excess weight or/and central obesity shown as hazard ratios (HR) with 95% confidence intervals (CI). Model 1 = crude, model 2 = adjusted for age at baseline, sex, maternal socioeconomic status, sweet treat and plant consumption at baseline, physical activity at baseline, and caries experience or periodontal health status, respectively

|  |  | **Model 1** | | | **Model 2** | | |
| --- | --- | --- | --- | --- | --- | --- | --- |
|  |  | **HR** | **95% CI** | | **HR** | **95% CI** | |
| **Excess weight** | |  | Lower limit | Upper limit |  | Lower limit | Upper limit |
|  | Caries experience vs. healthy | 1.28 | 0.84 | 1.95 | 1.34 | 0.88 | 2.05 |
|  | Gingivitis/calculus vs. healthy | 1.26 | 0.86 | 1.86 | 1.26 | 0.86 | 1.86 |
|  | Burden of oral diseases vs. healthy | |  |  |  |  |  |
|  | Caries OR gingivitis/calculus | 1.29 | 0.84 | 1.99 | 1.32 | 0.86 | 2.04 |
|  | Caries AND gingivitis/calculus | 1.58 | 0.90 | 2.80 | 1.67 | 0.94 | 2.97 |
| **Central obesity** | |  |  |  |  |  |  |
|  | Caries experience vs. healthy | 1.01 | 0.64 | 1.61 | 1.05 | 0.66 | 1.68 |
|  | Gingivitis/calculus vs. healthy | 1.39 | 0.92 | 2.08 | 1.33 | 0.88 | 2.00 |
|  | Burden of oral diseases vs. healthy | |  |  |  |  |  |
|  | Caries OR gingivitis/calculus | 1.34 | 0.85 | 2.12 | 1.30 | 0.82 | 2.07 |
|  | Caries AND gingivitis/calculus | 1.43 | 0.82 | 2.49 | 1.49 | 0.85 | 2.62 |
| **Excess weight and/or central obesity** | |  |  |  |  |  |  |
|  | Caries experience vs. healthy | 1.22 | 0.83 | 1.79 | 1.24 | 0.84 | 1.83 |
|  | Gingivitis/calculus vs. healthy | 1.28 | 0.91 | 1.82 | 1.27 | 0.89 | 1.80 |
|  | Burden of oral diseases vs. healthy | |  |  |  |  |  |
|  | Caries OR gingivitis/calculus | 1.29 | 0.88 | 1.90 | 1.30 | 0.88 | 1.92 |
|  | Caries AND gingivitis/calculus | 1.53 | 0.91 | 2.56 | 1.55 | 0.92 | 2.62 |

**Table S2** Characteristics of participants with oral health data available. ‘Included’ refers to participants in this study’s sample and ‘excluded’ to participants who did not participate in the follow-up data collection or had missing values in anthropometrics or questionnaire data (total n = 6660)

|  | **Included**  (n = 2702) | | **Excluded**  (n = 3958) | | *p*^a^ |
| --- | --- | --- | --- | --- | --- |
| Sex, n (%) |  |  |  |  | 0.090 |
| Girl | 1443 | (53.4) | 2030 | (51.3) |  |
| Boy | 1259 | (46.6) | 1928 | (48.7) |  |
| missing | 0 | | 0 | |  |
| Maternal SES, (%) |  |  |  |  | < 0.001 |
| Upper-level employee | 935 | (34.6) | 970 | (27.4) |  |
| Lower-level employee | 1069 | (39.6) | 1387 | (39.2) |  |
| Manual worker | 257 | (9.5) | 478 | (13.5) |  |
| Student | 255 | (9.4) | 412 | (11.7) |  |
| Other | 186 | (6.9) | 287 | (8.1) |  |
| missing | 0 | | 424 | |  |
| Weight status, n (%) |  |  |  |  | < 0.001 |
| Thin | 354 | (13.1) | 380 | (10.4) |  |
| Normal weight | 1997 | (73.9) | 2663 | (73.2) |  |
| Overweight | 294 | (10.9) | 489 | (13.4) |  |
| Obesity | 57 | (2.1) | 107 | (2.9) |  |
| missing | 0 | | 319 | |  |
| Central obesity, n (%) |  |  |  |  | < 0.001 |
| No | 2490 | (92.2) | 3282 | (89.3) |  |
| Yes | 212 | (7.8) | 393 | (10.7) |  |
| missing | 0 | | 283 | |  |
| Caries experience, n (%) |  |  |  |  | < 0.001 |
| No | 1992 | (73.7) | 2563 | (64.8) |  |
| Yes | 710 | (26.3) | 1395 | (35.2) |  |
| missing | 0 | | 0 | |  |
| Periodontal health status, n (%) |  |  |  |  |  |
| Good | 840 | (31.1) | 1199 | (30.3) | 0.489 |
| Gingivitis/calculus | 1862 | (68.9) | 2759 | (69.7) |  |
| missing | 0 | | 0 | |  |
| Burden of oral diseases, n (%) |  |  |  |  |  |
| Healthy | 1510 | (55.9) | 2112 | (53.4) | < 0.001 |
| Caries experience or gingivitis/calculus | 661 | (24.5) | 825 | (20.8) |  |
| Caries experience and gingivitis/calculus | 531 | (19.7) | 1021 | (25.8) |  |
| missing | 0 | | 0 | |  |

^a^ Results from Pearson’s chi-square test
